# Collagen mimetic peptides as novel therapeutics for vascular disease in the central nervous system

**DOI:** 10.3389/fnins.2025.1569347

**Published:** 2025-05-12

**Authors:** Olivia L. Bossardet, Joseph M. Holden, Brian J. Del Buono, Eric Schlumpf, Lauren K. Wareham, David J. Calkins

**Affiliations:** ^1^Department of Ophthalmology and Visual Sciences, Vanderbilt Eye Institute, Vanderbilt University Medical Center, Nashville, TN, United States; ^2^Sailfish Therapeutics, LLC, Stuart, FL, United States

**Keywords:** vascular dysfunction, cerebrovascular disease, neurodegeneration, collagen, collagen mimetic peptides, extracellular matrix, atherosclerosis, diabetes

## Abstract

**Background:**

Loss of vascular integrity is a common comorbidity of neurodegenerative diseases of the central nervous system (CNS). Compromised blood flow to the brain and excessive vascular remodeling is evident in chronic systemic cardiovascular diseases such as atherosclerosis, driving neurodegeneration and subsequent cognitive decline. Vascular remodeling occurs in response to changes in the microenvironment, with the extracellular matrix (ECM) as a major component. Collagens within the ECM and vascular basement membrane are integral to endothelial cell (EC) function and maintenance of the blood-brain barrier. Disruption of the ECM and breakdown of collagen with disease may lead to vascular dysfunction and neurodegeneration.

**Methods:**

We induced hyperglycemia in ApoE-deficient (*ApoE−/−*) mice by intraperitoneal injection of streptozocin (STZ; 50 mg/Kg) for 5 days and accelerated diabetic atherosclerotic disease through a high fat diet (HFD). Over a 12 weeks period, mice received weekly intravenous treatment of collagen mimetic peptide (CMP) or vehicle (phosphate buffered saline) to assess efficacy in promoting vascular integrity in central brain structures.

**Results:**

Following the STZ/HFD regimen, diabetic atherosclerotic *ApoE−/−* mice treated with CMP exhibited increased vascular integrity compared to vehicle in the cortex and in the CA1 and dentate gyrus regions of the hippocampus, as assed by higher levels of the endothelial cell adhesion glycoprotein CD31 and intravascular collagen IV, increased vascular area, and diminished leakage. Interestingly, in hippocampus, astrocytes were closer in proximity to vessels despite being less numerous in the CMP group.

**Conclusion:**

Collagen integrity is important for maintaining cerebrovascular architecture in disease. Application of CMP which intercalates with and repairs damaged collagen may have therapeutic use in neurodegenerative diseases by preserving vasculature structure and promoting blood-brain barrier integrity. These findings underscore the need to further explore the role of collagen repair as a novel therapeutic for diseases of the brain involving vascular degradation.

## 1 Introduction

Neurodegenerative diseases of the central nervous system (CNS) are often associated with cerebrovascular dysfunction. Compromised blood flow to the brain occurring as a result of vascular remodeling, often due to conditions such as hypertension and atherosclerosis, accelerates cognitive decline and the progression of neurodegenerative diseases such as Alzheimer’s disease and dementia (Ritson et al., 2024). Vascular remodeling is an adaptive process that occurs in response to both physiological and pathophysiological changes in the vascular microenvironment (Ma et al., 2020). The vascular microenvironment comprises a variety of extracellular matrix (ECM) proteins and ECM-degradative proteases, which act in concert to regulate cellular physiological and pathophysiological processes (Ma et al., 2020). Integral to the ECM are collagenous proteins which provide structure and stability to a wide range of tissues, including the vasculature (Wareham et al., 2024). Collagens I and III are most abundant in the vascular wall, while collagen IV is the main component of the basement membrane – an integral part of the blood-brain-barrier (Thomsen et al., 2017; Wareham et al., 2024). Endothelial cells (ECs) line the inner surface of vessels and serve as the barrier between the blood and vascular wall. ECs become dysfunctional in early vascular disease, instigating large-scale vascular remodeling that sometimes precedes eventual neurodegeneration in the CNS (Evans et al., 2022; Ritson et al., 2024). Vascular remodeling also characterizes atherosclerosis, the most common cause of cardiovascular disease worldwide (van Varik et al., 2012). Remodeling in atherosclerosis is associated with disruptions in collagen within the ECM (Evans et al., 2022). In the late stages of atherosclerosis, vascular cells including ECs secrete increased levels of matrix metalloproteases (MMPs), resulting in the proteolytic cleavage of collagen and destabilization of atherosclerotic plaques, thereby risking vessel rupture and thrombus formation (Airhart et al., 2014). Finally, patients with diabetes mellitus often exhibit accelerated atherosclerosis progression due to early EC damage and dysfunction (Grundy et al., 1999; Grundy et al., 2002).

Our recent research has highlighted the potential use of collagen mimetic peptides (CMPs) to repair collagen and counteract certain neurodegenerative processes (Ribeiro et al., 2022; Wareham et al., 2023; Wareham et al., 2024). These peptides interact with and anneal to damaged collagen to restore the helical structure of this important ECM component (Chattopadhyay et al., 2012; Chattopadhyay and Raines, 2014; Chattopadhyay et al., 2016). Here, we conducted a small study to explore the use of a CMP in preserving vascular architecture in systemic cardiovascular disease using an established model of atherosclerosis that uses streptozocin (STZ) to accelerate EC dysfunction in *ApoE−/−* (apolipoprotein E) mice maintained on a high-fat diet (Tucureanu et al., 2019; Ganbaatar et al., 2020). This mouse strain is the most widely used for preclinical studies of atherosclerosis. Deficiency in endogenous ApoE leads to cytokine and protease secretion with subsequent inflammation and extracellular matrix degradation (Bink et al., 2013; Lo Sasso et al., 2016), while systemic STZ application induces a form of type I diabetes through reduced endothelium-dependent vasodilator response and destruction of pancreatic islet β-cells (Ding et al., 2005; Furman, 2021). We found that systemically administered CMP vs. vehicle stabilizes vascular structure in three distinct brain regions. Furthermore, in the CA1 of the hippocampus, CMP increases astrocyte proximity to vessels. These findings suggest possible benefits of using CMPs to repair collagen in and around the vascular bed as a novel therapeutic for vascular-related neurodegenerative disease.

## 2 Materials and methods

### 2.1 Animals and study design

All animal studies were conducted in accordance with the NIH guide for the care and use of laboratory animals and approved by the Vanderbilt University Institutional Animal Care and Use Committee. For the studies described, 12 weeks-old ApoE−/− (#002052) on C57/B6J wildtype (WT) background were obtained from Jackson Laboratories. Mice were housed in a facility managed by Vanderbilt University Division of Animal Care with ad libitum access to water and standard diet followed by ad libitum access to 45% high fat diet (Research Diet Inc.; Catalog #D12451) starting at the correct study time point. Mice were subjected to 12 h. light/dark cycle.

Prior to STZ injection and diet change, baseline fasting blood glucose measurements and weights were obtained. To induce hyperglycemia *ApoE*−*/*− mice were injected with STZ (50 mg/Kg in 10 mM sodium citrate pH4.5; Millipore Sigma Catalog #S0130) intraperitoneally for five consecutive days. For the duration of STZ injections, mice received drinking water supplemented with 10% sucrose. One week after the final STZ injection, blood glucose levels were measured to confirm hyperglycemia. Any mice not registering as hyperglycemic were removed from the study. Remaining mice were then switched to a 45% high fat diet (HFD) to accelerate atherosclerotic plaque formation. After 1 week on HFD, mice were randomly assigned to receive intravenous CMP or vehicle (1x phosphate buffered saline, PBS), once weekly for 12 weeks.

Mouse body weights were taken at baseline (before STZ treatment) and once per week for the duration of the study. Mouse blood glucose measurements were taken at the same time intervals using human monitoring test strips (CareTouch). Mice were fasted for 6 h prior to glucose measurement and then anesthetized with 2.5% isoflurane prior to microsyringe withdrawal of 10–15 μl of blood. Any residual bleeding was stemmed using a silver nitrate applicator (Avoca reference number# 7482) before returning mice to their cages.

### 2.2 Treatment cohorts: intravenous administration

The particular collagen mimetic peptide (CMP) used is a 21-residue single-strand peptide consisting of a seven-repeat sequence of hydroxyproline (Hyp), glycine (Gly), and 4-fluoroproline (Flp), abbreviated as (*cis*-Flp-Hyp-Gly)_7_, manufactured using standard solid-phase peptide synthesis chemistry in limited quantity by Bachem, AG (Germany) and described as CMP13A in our previous studies (Bou Ghanem et al., 2023). CMP was dissolved in sterile 1X PBS at a concentration of 1 mg/Kg and sterilized using a 0.22 μm filter (Millipore Sigma, Burlington, MA, United States). Mice were weighed and restrained in an apparatus designed for tail vein injection. Tails were heated to dilate the veins prior to weekly injection, and 100–150 μL of CMP13A in vehicle (1x PBS) was injected to accommodate a final concentration in each animal of 0.1 mg/Kg. Our sample size for weight and blood glucose measurements included 13 mice in the vehicle (PBS) group (seven males, six females) and 14 in the CMP group (six males, eight females).

An additional batch of this CMP was produced with attachment of the Tide Fluor™ 2 moiety (AAT Bioquest, Sunnyvale, CA, United States) as a fluorescent reporter. This was injected intravenously at 100 μM concentration (100 μL) to identify binding in *ApoE−/−* + STZ mice. This CMP was injected once, 48 h prior to sacrifice and again immediately before transcardial perfusion of 4% PFA to assess localization of the CMP in the CNS. Finally, 1 day prior to sacrifice, a subset of mice was restrained in tail vein apparatus and injected intravenously with 100 μL Sulfo-NHS biotin (“biotin”) in sterile 1X PBS (Thermo-Fisher; Catalog #21335), following published protocols (Ding et al., 2023). Tissues of interest were mounted on microscope slides with DAPI Fluoromount-G counter stain (Catalog #0100-20; Southern Biotech, Birmingham, AL, United States) for imaging.

#### 2.3 Immunohistochemistry

Upon conclusion of the study, mice were anesthetized via intraperitoneal pentobarbital injection followed by transcardial perfusion of 1X PBS and 4% paraformaldehyde (PFA). Perfused brains were removed from 4% PFA after 24 h and cryoprotected in a sucrose gradient series (20%–30%). Brains were cut into 50 μm sections using a freezing sliding microtome (SM2000R; Leica Biosystems, Buffalo Grove, IL, United States), and slices containing hippocampus were identified for immunolabeling. Tissue was cryopreserved by sucrose gradient and underwent multiple freeze-thaw cycles for permeabilization. Sections were washed for 10 min in 1X PBS and further permeabilized in 0.5% Triton X-100 in 1X PBS overnight at 4°C. Sections were blocked at room temperature (5% normal donkey serum in 0.1% Triton X-100 in 1X PBS) for 2 h while shaking, followed by primary antibody incubation. Antibodies against CD31 (rat 1:200; BD Pharmingen, 550274), collagen IV (goat 1:100, EMD Millipore AB769), and GFAP (goat 1:200; Abcam, Cambridge, United Kingdom, Catalog #ab53554) or isolectin GS-IB4 biotin-XX conjugate (1:200; Invitrogen; Waltham, MA, United States, Catalog #I21414) in 3% normal donkey serum in 1X PBS and 0.1% triton were incubated at 4C for 3–5 days while rocking. Sections were washed with 1X PBS and placed in secondary antibody solution (1% donkey serum and 0.1% Triton in 1X PBS) containing secondary antibodies: donkey anti-rat Alexa 488 (1:200; Jackson Immuno Research; 712-545-150), donkey anti-goat Alexa 555 (1:200; Jackson Immuno Research; Catalog #705-565-147), and Strepdavadin Alexa-647 (1:200; Invitrogen; Catalog #S21374), an antibody that binds to Sulfo-NHS Biotin for fluorescent visualization. Sections were incubated for 2 h at room temperature before washing in 1X PBS. Sections were mounted on microscope slides (Diamond White Glass Microscope Slides; White frosted; #1358W) with DAPI Fluoromount-G (Catalog #0100-20, Southern Biotech, Birmingham, AL, United States) and cover slipped (microscope cover glass; Globe; #1414-0) sealed with nail polish (Ted Pella Inc; #114-7) for imaging.

### 2.4 Imaging and quantification

Fluorescent images of the brain were taken on Nikon Ti-E Spinning Disk confocal microscope. For the brain, 10x montages were taken to identify the hippocampus CA1 region, dentate gyrus (DG), and cerebral cortex; region-specific images were then acquired at 20 × or 60 × magnification. Z-stacks of equal thickness were taken at a step size of 0.9 μm or 0.3 μm respectively depending on magnification, which were combined using the standard deviation Z-stack option in Fiji ImageJ.

Mean intensities of GFAP, CD31, collagen IV, and biotin immunofluorescence in the CA1 region, DG, and cortex were obtained using the measure tool in Fiji software. CD31 images were passed through an intensity threshold filter and binarized. The ImageJ “Particle Analysis” 30-infinity tool was then used to find blood vessels and capture shape descriptor information regarding area and other parameters not reported here. The distance from GFAP-labeled astrocytes to vessels was measured in images of CA1 and DG brain regions. Cortex was not included in the analysis due to sparse astrocyte density. The “Particle Analysis” 30-infinity tool was used to find individual astrocytes bodies and their centroid was measured. Using a custom Python script, the minimum distance from each centroid to the closest blood vessel was recorded. To obtain an estimate of leakage of injected fluorescent biotin, images from cortical sections were analyzed using Fiji ImageJ and a custom Python script. The biotin channel was projected to max intensity Z-projection. The background fluorescence was removed by subtracting a large (64-pixel radius) Gaussian-filtered copy from each image. Blood vessels were then segmented by analyzing the “tubeness” (ImageJ plugin) of the image with a sigma of 2.0, and the resulting image passed through an Auto “Li” Threshold that preserved as much of the information as possible to produce a binary mask. This mask was applied to the background-reduced image, removing the vessels and leaving any excess biotin in the tissue space to produce a map detailing the distance from each pixel to a segmented vessel. This map, combined with the vessel-removed image, allowed us to set an upper limit of distance to nearest vessel of 15 pixels (∼7 μm), and calculate with a Python script the number and intensity of all positive (non-zero) biotin pixels in that range.

### 2.5 Statistical analyses

Statistics were conducted in GraphPad Prism version 10.1.2. After testing for normality, ordinary one-way ANOVA, Brown-Forsythe one-way ANOVA, and non-parametric tests including Kruskal Wallis, Mann Whitney, and Welch’s were used. All quantification was performed on multiple sections through each brain (with sample size given in legends) using 60 × magnification confocal images from each.

## 3 Results

### 3.1 Weight and blood glucose levels in ApoE−/− STZ mice with HFD

**FIGURE 1:**
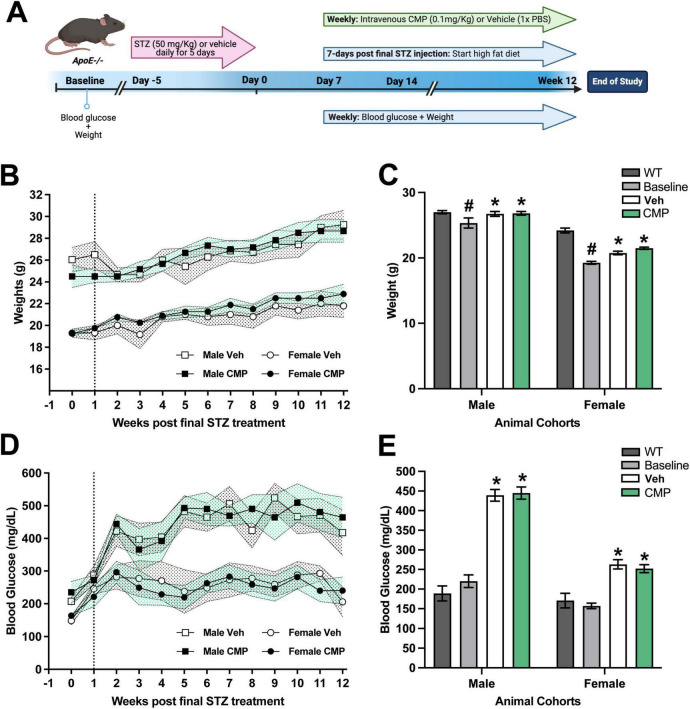
Blood glucose and body weight were not affected by treatment regimen in mouse model (*ApoE−/−* + STZ). **(A)** ApoE knockout animals (*ApoE−/−*) were used for the atherosclerosis-diabetes-high fat model. Baseline weights and blood glucose measurements were taken 5 days before intraperitoneal streptozocin (STZ) (50 mg/Kg) injection. On day 7 post-final STZ injection blood glucose and weights were measured. Once hyperglycemia was confirmed, animals were switched to a high-fat diet (HFD; 45% fat). At initiation of HFD mice were randomly assigned to receive once weekly intravenous collagen mimetic peptide (CMP) (CMP13A, 0.1 mg/Kg) or vehicle control (1x PBS) and blood glucose and weight measurements for the remainder of the study. At 12 weeks post-STZ, animals were euthanized, tissue processed, and endpoints measured. **(B)** Mean body weight for *ApoE−/−* males and females increased over time following initiation of HFD at week 1 (dashed line) for both vehicle and CMP cohorts with considerable overlap in standard error of the mean (Veh: stippled gray; CMP: stippled green). **(C)** Compared to wildtype (WT, *n* = 2), at week 0 (baseline) *ApoE−/−* males and females were significantly lower in weight (#*p* < 0.0001). Following the experimental period, mean weight increased from baseline after HFD in both vehicle and CMP groups for males (*p < 0.0001) and females (*p ≤ 0.005). The weights of mice did not differ between vehicle and CMP groups for either males or females for the duration of the study (*p* ≥ 0.11). **(D)** Mean blood glucose levels for *ApoE−/−* males and females also increased over time with overlap in standard error of the mean (Veh: stippled gray; CMP: stippled green). **(E)** Blood glucose levels at baseline (day 0) for *ApoE−/−* males and females did not differ from WT (*p* > 0.05). Following the experimental period, glucose for males and females increased in both PBS and CMP groups compared to baseline (*p < 0.001). For either sex, there was no significant difference between vehicle and CMP groups (*p* ≥ 0.36). Cohorts: for vehicle, seven males, six females; for CMP, six males, eight females. For all comparisons, data presented as mean ± SEM. Statistical analyses = Two-way ANOVA, Tukey’s multiple comparisons test.

A diabetic atherosclerosis mouse model was implemented by combining *ApoE*−*/*− mice with a streptozocin STZ-induced diabetic phenotype (Figure 1A). To determine whether STZ treatment led to hypoglycemia, we measured fasting blood glucose along with body weight at baseline (day 0) and then weekly for the duration of the study. For both vehicle- and CMP-treated *ApoE*−*/*− cohorts, weight steadily increased over the 12 weeks period after being placed on HFD (Figure 1B), though males were consistently about 25% heavier. Compared to WT mice, *ApoE*−*/*− mice had a lower average weight at baseline for both males and females (Figure 1C; *P* < 0.001). Average baseline weight for males exceeded that of females as expected in both WT and *ApoE*−*/*− mice (24.8 vs. 19.3 g and 27.0 vs. 24.2 g, respectively; *p* < 0.001), and average weight for both increased after treatment compared to baseline in both vehicle- and CMP-treated cohorts (*p* ≤ 0.005). There was no significant difference in weight between vehicle and CMP cohorts for either sex (*p* ≥ 0.11). Similarly, blood glucose levels also remained elevated following the period of STZ treatment for both males and females (Figure 1D). Average blood glucose levels were higher in males compared to females at baseline: 187.9 and 157.1 mg/dL, respectively (*p* = 0.009; Figure 1E). There was no significant difference between average blood glucose in WT vs. *ApoE*−*/*− mice in either sex at baseline (*p* > 0.05). After STZ and HFD, average blood glucose levels increased in both the vehicle- and CMP-treated mice for both sexes compared to baseline across the 12 weeks period (*p* < 0.0001). There was no significant difference in blood glucose between vehicle- and CMP-treated cohorts for either sex (*p* ≥ 0.36).

### 3.2 Vascular collagen IV increases with CMP treatment

**FIGURE 2:**
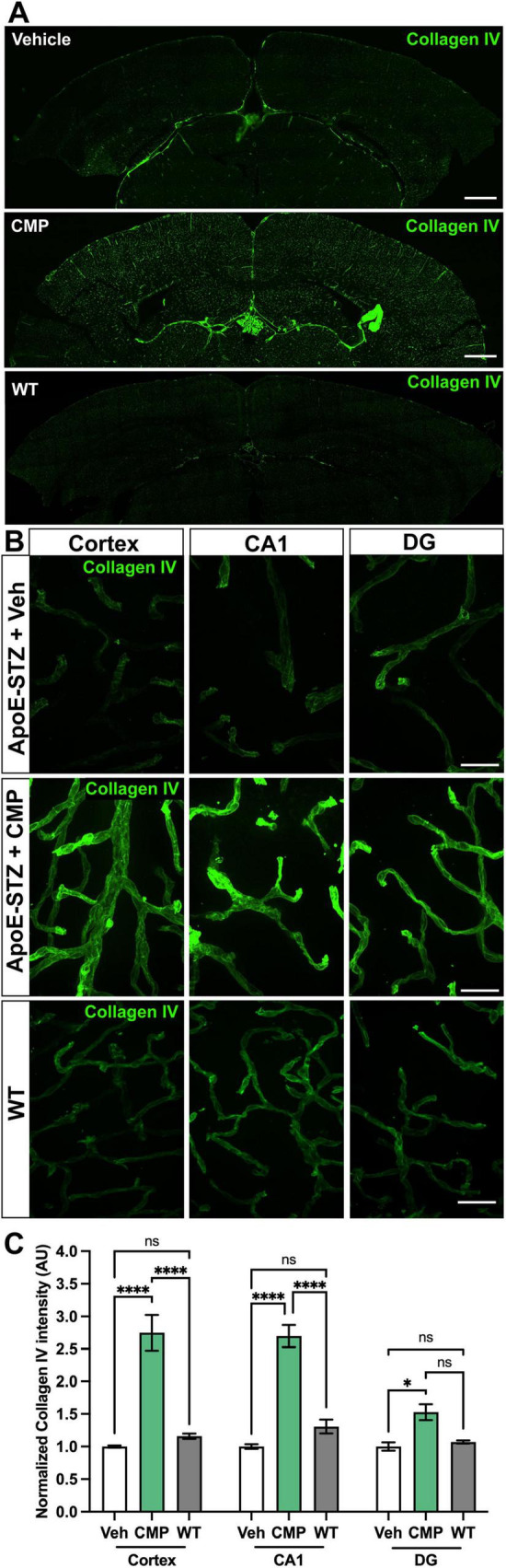
Collagen IV vascular labeling in the brain is increased after collagen mimetic peptide (CMP) treatment. **(A)** Representative montage of confocal images of collagen IV immunolabeling in the brain of vehicle-treated and CMP-treated *ApoE−/−* mice with wildtype (WT) shown for comparison. Scale bar = 500 μm. **(B)** Representative high-magnification confocal images of collagen IV immunolabeling in the cortex and CA1 and dentate gyrus (DG) areas of the hippocampus in vehicle-, CMP-treated, and WT mouse. **(C)** Quantification of collagen IV intensity (normalized to vehicle). CMP treatment significantly increased collagen IV intensity in all areas compared to vehicle (*p = 0.04, ****p < 0.0001), which was slightly less than WT (ns, *p* > 0.05). Two-way ANOVA Tukey’s multiple comparisons tests. *n* = 9 sections for CMP, seven for vehicle, and four for WT. Data presented as mean ± SEM. Scale = 30 μM.

Collagen IV is the primary component of the vascular basement membrane, an integral part of the blood-brain-barrier that adds stability to blood vessels (Paulsson, 1992; Marchand et al., 2019). Levels of collagen IV assessed by immunolabeling appeared higher in the CMP-treated cohort vs. vehicle and WT for all three brain regions as shown in montage images (Figure 2A). In fact, detection of collagen IV in vehicle-treated animals was difficult, especially in the cortex and CA1 region of the hippocampus (Figure 2B). When quantified, CMP treatment increased collagen IV intensity in *ApoE*−*/*− mice by 175%, 170%, and 52% in cortex, CA1, and DG, respectively, relative to levels seen in the corresponding regions in vehicle animals (Figure 2C). Levels of collagen IV did not significantly differ between WT and vehicle-treated animals across all regions. These data support the idea that CMP enhances levels of basement membrane collagen IV, which may be beneficial for improving vascular integrity.

### 3.3 CMP increases endothelial CD31

**FIGURE 3:**
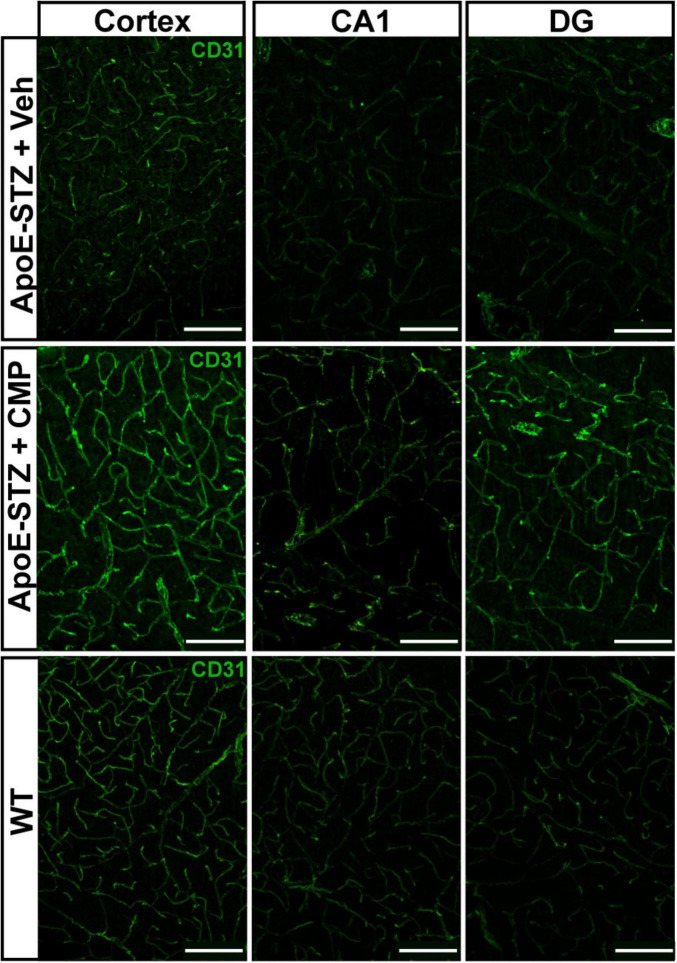
CD31 vessel area and intensity in the brain is increased after collagen mimetic peptide (CMP) treatment. Representative confocal images of CD31 immunolabeling in the cortex, CA1 and dentate gyrus (DG) in vehicle, CMP, and wildtype (WT). Scale = 100 μM.

**FIGURE 4:**
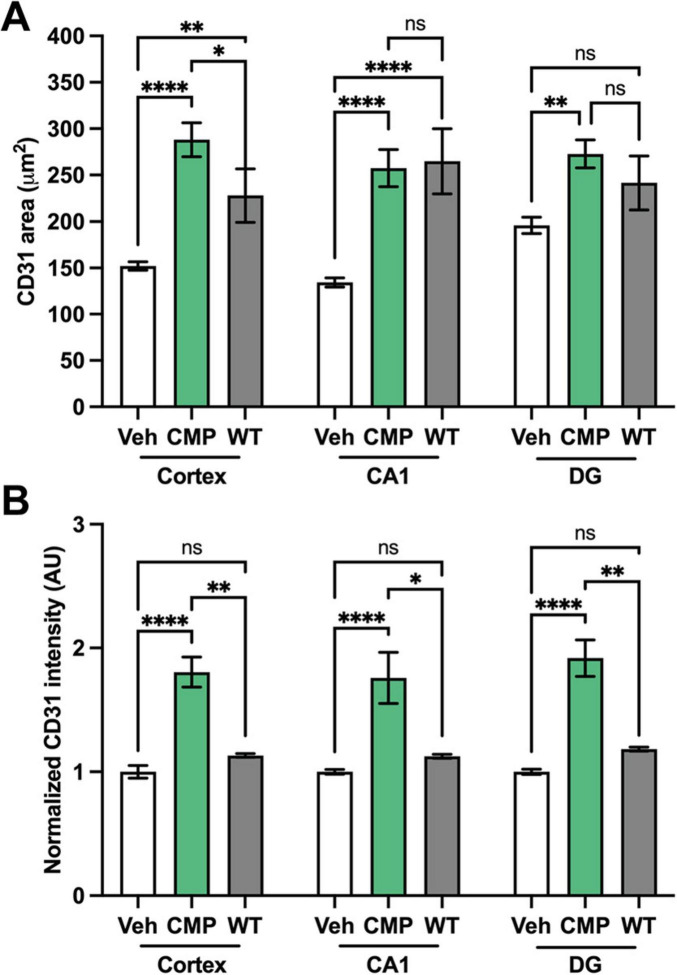
Quantification of CD31 area and intensity in cortex, CA1, and dentate gyrus (DG). **(A)** Mean area of CD31+ vessel segments decreased in vehicle animals compared to wildtype (WT) in cortex and CA1 (**p = 0.002; ****p < 0.0001), but not DG (ns, *p* = 0.06). With collagen mimetic peptide (CMP) treatment, CD31 area significantly increased in cortex, CA1, and DG regions relative to vehicle (**p = 0.03; ****p < 0.0001) and relative to WT in the cortex (*p = 0.03) while reaching WT levels in CA1 and DG. **(B)** Mean CD31 intensity in vessel segments (normalized to vehicle) was not changed between vehicle-treated and WT animals (*p* > 0.05). However, CMP increased CD31 intensity beyond vehicle-treated or WT in all brain regions (*p = 0.01; **p = 0.007; ****p < 0.0001). *n* ≥ 1,021 vessel segments for CMP, ≥ 1,208 for vehicle, and ≥ 642 for WT across nine, seven, and fiur sections, respectively. Two-way ANOVA Tukey’s multiple comparisons test for statistical analyses. Data presented as mean ± SEM.

To further assess vascular structure and EC density, we immunolabeled brain sections against the endothelial cell adhesion glycoprotein CD31, an effective EC and vascular marker (Liu and Shi, 2012; Cheung et al., 2015; Cheung et al., 2020). CD31 labeling was more pronounced in *ApoE*−*/*− mice with CMP treatment compared to vehicle and WT mice in all brain regions, indicating improved vascular density (Figure 3). Consistent with this observation, the mean area of individual CD31+ vessel segments increased in all brain regions of CMP-treated *ApoE−/−* mice compared to vehicle (Figure 4; **p = 0.02, ****p < 0.001), and CD31+ area was increased beyond levels seen in WT cortex (*p = 0.03). Finally, CD31 label intensity averaged from measurements in individual vessel segments independent of their area increased in CMP-treated mice compared to vehicle and WT animals in all three brain regions *p = 0.01, **p = 0.007, **** < 0.0001).

### 3.4 CMP promotes astrocyte-vascular interactions in the brain

**FIGURE 5:**
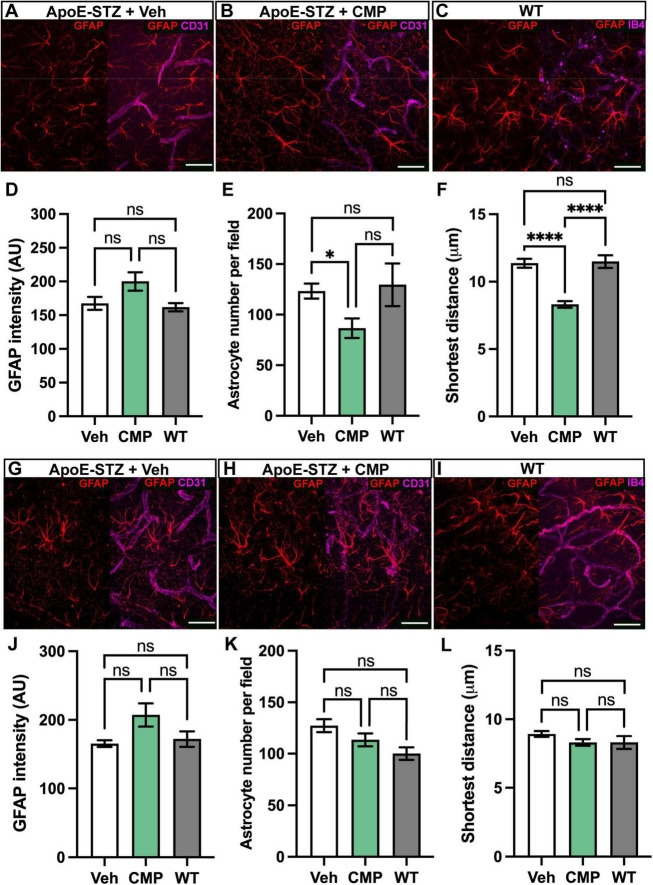
Astrocyte-vessel interactions in the hippocampus. **(A–C)** Representative confocal images of glial fibrillary acidic protein (GFAP)-positive astrocytes with immunolabeling against a vascular marker, either CD31 as in **(A)** and **(B)**, or Isolectin-B4 (IB4) as in **(C)**, in CA1 region of hippocampus of **(A)** vehicle-, **(B)** collagen mimetic peptide (CMP)-treated, and **(C)** wildtype (WT) mouse. Scale = 30 μM. **(D)** The minor difference in GFAP intensity between CMP- and vehicle-treated and WT groups was not significant (ns; *p* > 0.05). **(E)** The number of astrocytes per equally-sized image field in the CA1 was less in CMP-treated mice (*p = 0.01) but not significantly different to WT (*p* > 0.05). **(F)** The shortest distance from the centroid of a given astrocyte to the nearest blood vessel was less in CMP-treated mice (*n* = 1,156 astrocytes measured) compared to vehicle-treated (*n* = 1,035 astrocytes) and WT mice (*n = 518* astrocytes; ****p < 0.0001). **(G–I)** Representative confocal images of GFAP-labeled astrocytes with immunolabeling against a vascular marker, either CD31 as in **(G)** and **(H)**, or Isolectin-B4 (IB4) as in **(I)**, in DG region of hippocampus of **(A)** vehicle-, **(B)** CMP-treated, and **(C)** WT mouse. Scale = 30 μM. **(J)** GFAP intensity was again slightly higher with CMP treatment but not significant (*p* > 0.05). **(K)** The number of astrocytes per image in the DG region was similar in all groups (*p* > 0.05). **(L)** The shortest distance from the centroid of a given astrocyte to the nearest blood vessel not significantly different across all groups (*p* > 0.05; vehicle *n* = 1,527, CMP *n* = 1,360, and WT *n = 300* astrocytes measured. All data are presented as mean ± SEM. Two-way ANOVA Tukey’s multiple comparisons test for statistical analyses.

To evaluate the impact of CMP on glial-vascular interactions in CA1 and DG, we quantified astrocyte number and proximity to blood vessels in CMP- and vehicle-treated *ApoE−/−* mice and WT mice using immunolabeling against glial fibrillary acidic protein (GFAP). The cortex was excluded due to insufficient signal in GFAP staining. Representative images of astrocytes and their vessel interactions are shown in Figure 5. GFAP intensity was not significantly different across groups in the CA1 (Figures 5A–F; *p* > 0.05). Next, we identified individual astrocyte cell soma; the number of astrocyte soma was lower in the CA1 region of CMP-treated mice compared to vehicle-treated animals, but not different from WT (*p* = 0.01 and *p* > 0.05). Finally, using astrocyte soma coordinates, we calculated the distance to the nearest blood vessel in CMP-, vehicle-treated, and WT mice. Interestingly, despite fewer astrocytes in the CA1, in the CMP-treated group astrocytes were closer to vessels than in vehicle-treated or WT mice. In the DG region (Figures 5G–L), GFAP intensity was also not significantly different across groups (*p* > 0.05). In contrast to the CA1 the number of astrocyte soma in the DG remained unchanged between groups (*p* > 0.05). In the DG astrocyte soma distances to blood vessels were the same (*p* > 0.05).

### 3.5 CMP crosses the blood-brain-barrier to repair vessels and reduce leakage

**FIGURE 6:**
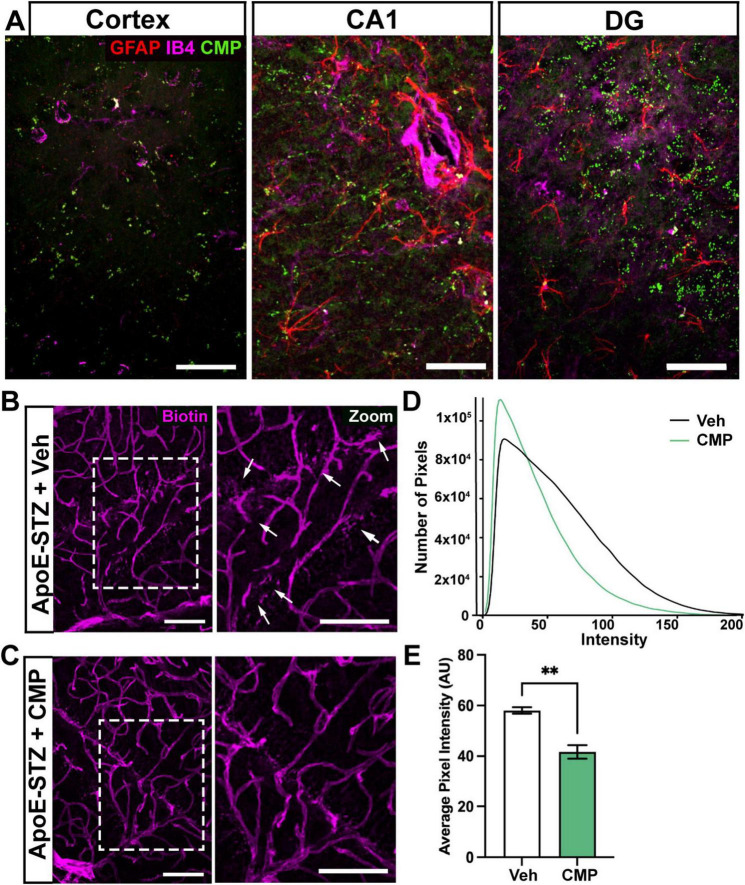
Collagen mimetic peptide (CMP) crosses the blood-brain barrier and reduces leakage. **(A)** Intravenous injection of a fluorescently labeled CMP (green) led to punctate binding in the cortex, CA1, and dentate gyrus (DG). Immunolabeling against glial fibrillary acidic protein (GFAP) (red) is shown with vasculature using IB4 (purple) to reveal local pockets of astrocyte processes and vessels, respectively, for comparison. Scale = 100 μM. **(B,C)** Representative confocal images of cortical sections immunolabeled against biotin following intravenous (tail-vein) injection of **(B)** vehicle- and **(C)** CMP-treated mice. Perivascular leakage of biotin was most evident in vehicle tissue (arrows, zoomed area on right) compared to CMP. Scale = 50 μM. **(D)** Histograms of number of biotin-fluorescing pixels (following background removal) in cortical sections within a criterion distance (15 pixels, ∼7 μm) of the nearest blood vessel. Vehicle tissue demonstrated greater high-intensity biotin signal compared to CMP; this is reflected in significant difference between the mean pixel intensity **(E)**, **, *p* = 0.0044; Welch’s *t*-test, for unequal variances. Data presented as mean ± SEM; *n* = 9 images for CMP; four for vehicle).

To test whether CMP crosses the blood-brain-barrier, we intravenously injected *ApoE−/−* STZ mice with the same CMP but with an attached fluorescent tag. Regions of punctate fluorescent CMP indicating areas of damaged collagen were apparent in the brain of *ApoE−/−* STZ mice, especially in the DG (Figure 6A). The pattern of CMP binding in the *ApoE−/−* STZ mouse was closely associated with reactive astrocytes and blood vessels. To assess whether CMP influences vascular leakage and vessel structure in the brain, we quantified fluorescent biotin content following intravenous injection of this tracer, which is often used to assess breakdown of the blood-brain-barrier (Ding et al., 2023). In vehicle-treated *ApoE−/−* mice, sections of cortex show perivascular clusters of biotin diffused from nearby vessels (Figure 6B). In CMP-treated mice, this tendency was greatly diminished, despite the higher density of the vasculature (Figure 6C). Using an algorithm that identified biotin-labeled pixels within a fixed distance of the nearest blood vessel, we found that cortex from vehicle-treated mice contained a greater number of pixels of higher intensity within this distance than cortex from CMP, where the biotin diffusion was far less intense (Figure 6D). This pattern is reflected in mean pixel intensity for the vehicle cohort, which was 45% greater than for the CMP cohort (Figure 6E).

**FIGURE 7:**
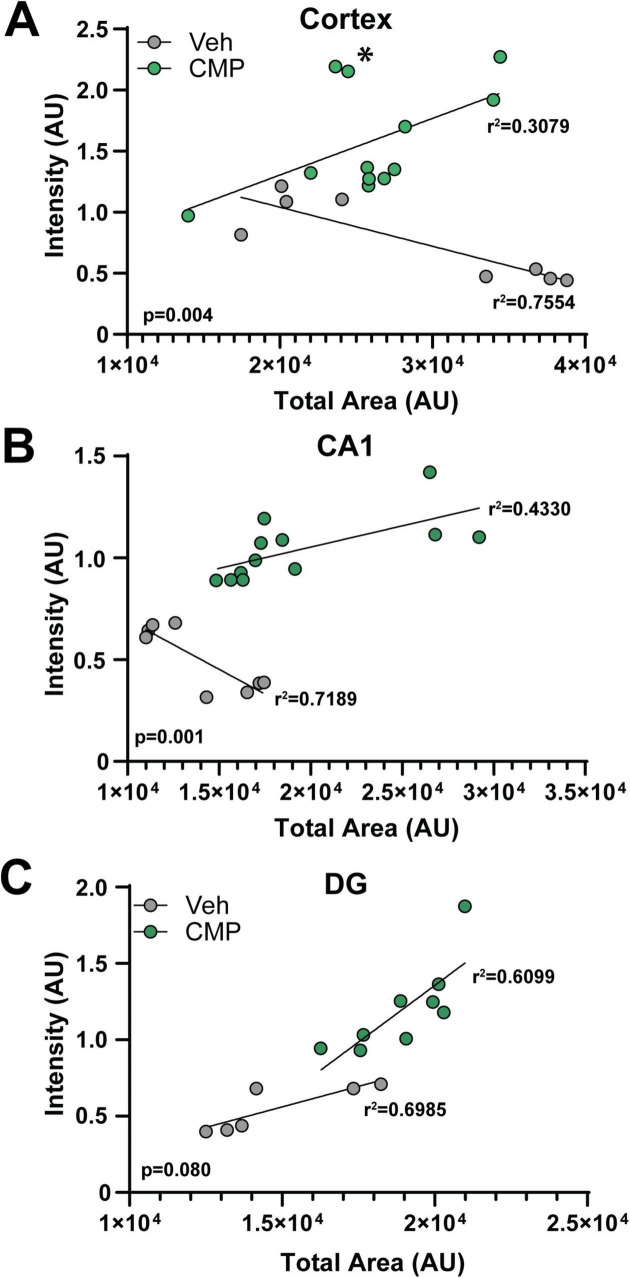
Collagen mimetic peptide (CMP) preserves vessel area with increased CD31. Total fluorescent intensity of CD31 immunolabel (in arbitrary units, AU) summed over the total area of CD31+ vessel segments in cortical **(A)**, CA1 **(B)**, and dentate gyrus (DG) **(C)** regions of the brain from vehicle- and CMP-treated *ApoE−/−* mice; Pearson correlation coefficients for the best-fitting linear regression are given for each set of data. For vehicle, CD31 intensity decreased in cortex and CA1 even as the area of CD31+ segments increased, indicating diminishing CD31 prior to vessel loss (*p* ≤ 0.038), while for CMP, the opposite occurred, reaching significance for CA1 and DG (*p* ≤ 0.013) but not in cortex (*p* = 0.061) due to outlying samples (indicated by *). For the cortex and CA1 region, the slopes of the regression lines for vehicle and CMP were significantly different (*p*-values indicated; Analysis of Covariance; *n* = 9–12 sections for CMP, 6–8 for vehicle).

Finally, the reduction in biotin diffusion suggests that increased CD31 immunolabel with CMP treatment, as shown earlier (Figures 3, 4), could be indicative of a vascular reparative effect rather than simply local increases in expression. To assess this possibility, we compared the total intensity of CD31 label summed over all vessel segments in an image to the total summed area of labeled vessels (Figure 7). For vehicle-treated *ApoE−/−* mice, in cortex and CA1, CD31 intensity diminished with increased area of labeled vessels; the opposite trend occurred in DG. In each case, the correlation was significant (*p* ≤ 0.038). For CMP-treated mice, for all three brain regions as vessel area increased, so did CD31 label, with significant correlation between the two variables for the hippocampus CA1 and DG (*p* ≤ 0.02). In cortex, the correlation was nearly significant (*p* = 0.061), with two samples indicating higher than expected CD31 for a given total vessel area. These data indicate that increased CD31 intensity is associated with expansion of vessel area with CMP treatment.

## 4 Discussion

In our previous work, we demonstrated the potential of CMP repair of collagen to counteract neurodegeneration in the optic nerve projection to the brain (Ribeiro et al., 2022; Wareham et al., 2023; Wareham et al., 2024). Diabetes and atherosclerosis are systemic diseases associated with age-related neurodegenerations of the CNS including Alzheimer’s disease, diabetic retinopathy, and dementia (Baradaran et al., 2020; Gasecka et al., 2020; Cicolari et al., 2021; Soni et al., 2021). Since there is an emergent role of vascular dysfunction as an early driver of neurodegeneration (Bou Ghanem et al., 2024), we explored whether CMPs have the potential to preserve cerebrovascular architecture in a mouse model of accelerated diabetic atherosclerotic disease: *ApoE−/−* STZ mice fed a HFD (Park et al., 1998; Ding et al., 2005; Park et al., 2016).

Mice with ApoE deficiency fed a standard diet exhibit an imbalance of cholesterol in macrophages, causing high levels of cytokine and protease secretion, and triggering inflammation and ECM degradation (Lo Sasso et al., 2016). Feeding *ApoE−/*− mice a high fat diet leads to an acceleration of atherosclerotic plaque formation and widespread inflammation, with increased adhesion molecule expression and leukocyte recruitment, as well as degeneration of the microvessels and a reduction in microvascular length (Bink et al., 2013). Changes in vessel structure correlated with activation of the proinflammatory cyclophilin A (CypA)–nuclear factor-κB–matrix metalloproteinase-9 (MMP9) pathway (Bell et al., 2012). Activation of MMP9 leads to the degradation of collagen in the ECM, which may promote inflammation and vessel degradation (Zhu et al., 2021). STZ is an antibiotic that causes pancreatic islet β-cell destruction, triggering hyperglycemia (Furman, 2021). When STZ is combined with ApoE deficiency, mice exhibit gross endothelial cell dysfunction (Ding et al., 2005).

To investigate the effect of CMP treatment on vascular pathology, we treated mice with intravenous vehicle or CMP for a period of 12 weeks and assessed vascular structures in the brain, comparing results to naïve WT animals (Figure 1). All *ApoE−/−* mice included in the study became hyperglycemic after STZ treatment compared to baseline measurements. Weights of all animals also increased similarly across both groups; CMP had no effect on weight gain or blood glucose levels compared to vehicle; males were larger and demonstrated higher levels of glucose compared to females. We found that CMP had a positive effect on preserving vascular density in the brain. Compared to vehicle-treated and even WT animals, levels of collagen IV (the primary component of the vascular basement membrane) increased (Figure 2). CMP treatment also elevated levels of CD31 localized to blood vessels in the cortex, CA1, and DG beyond WT levels and increased areas of vessels containing CD31 (Figures 3, 4). Since CD31 is a glycoprotein involved in endothelial cell adhesion in vasculature (Albelda et al., 1991), we hypothesize that CMP heals damaged collagen in the vessel basement membrane, thereby promoting endothelial cell survival and vessel integrity. CMP also promoted astrocyte-blood vessel interactions in the CA1 region of the hippocampus; astrocyte soma were closer in proximity to vessels after CMP treatment compared to vehicle and WT animals, even though there were fewer GFAP-labeled astrocytes (Figure 5). Finally, injection of fluorescent CMP confirmed that the mimetic peptide crossed the blood-brain barrier, reaching the cortex and hippocampus of the brain (Figure 6). ECM turnover is a dynamic but tightly controlled process; in homeostasis a balance between protein degradation and formation exists (Kehlet et al., 2018; Matsubayashi, 2022; Wareham et al., 2024). In disease however, the balance is disrupted with elevated levels of MMPs, leading to increased production of fragmented and degraded collagen with exposed ligand binding sites that may be involved in multiple pro-inflammatory pathways (Wareham et al., 2024).

When assessing the effect of CMP on vasculature in the brain, we first quantified collagen intensity, the primary component of the vascular basement membrane (Wareham et al., 2024). CMP increased collagen IV intensity beyond levels observed in vehicle-treated animals. Since CMP intercalates into damaged collagen strands to restore the native triple helical structure, it is not surprising that vessel expression of collagen IV was enhanced. However, what was most surprising was that CMP increased collagen IV intensity levels beyond those seen in healthy, WT animals. Since there are basal levels of collagen turnover in healthy tissue during homeostasis, it is possible that the lower collagen IV intensity observed in WT animals reflect a basal maintenance level of collagen IV. An interesting next step would be to assess whether the CMP effect on collagen IV changes vessel integrity; a future study involving a later timepoint in disease progression where blood-brain barrier breakdown in the model is apparent would be beneficial.

We next measured levels of the EC and vascular marker, CD31 (Lertkiatmongkol et al., 2016). CD31, or Platelet/Endothelial Cell Adhesion Molecule-1 (PECAM-1), is a cell adhesion molecule highly expressed on the surface of ECs, and to a lesser extent by a range of immune cells including monocytes, neutrophils, and certain T-cell subsets. CD31 is an integral component of the microvascular barrier, forming tight junctions at EC borders that mediate vascular permeability and leukocyte trafficking (Lertkiatmongkol et al., 2016). In the cortex and hippocampal areas of the brain that we explored, CD31 was elevated by CMP treatment relative to vehicle and WT animals. We detected a decrease in vascular density (as measured by labeled vessel area) in diseased animals compared to WT that appeared to be prevented with CMP treatment. CD31 is necessary for maintaining immune privilege of vascular endothelium and for preserving vascular structure and integrity (Cheung et al., 2015; Cheung et al., 2020). Thus, the improved vascular structure we observed may also be due to elevation of CD31 induced by CMP treatment, which again suggests a potential therapeutic benefit of CMP treatment in such contexts since preventing vascular breakdown is crucial in maintaining the blood-brain barrier and immune privilege of the brain to prevent neurodegeneration (Wareham et al., 2022). Consistent with this role, using fluorescent biotin to assess the blood-brain-barrier as described (Ding et al., 2023), we found that CMP reduced vascular leakage (Figure 6). That CD31 summed across vessel segments increased with total vessel area (Figure 7) suggests that enhanced levels were concomitant with vessel preservation with CMP treatment and not just reflective of localized increases.

While CMP increased CD31 in all areas tested, interestingly, in vehicle-treated animals CD31 levels persisted in the DG compared to the cortex and CA1 (Figure 4A; *P* = 0.06) and even increased with increasing vessel area in DG (Figure 7). In vascular dementia, ischemic injury is known to affect brain areas differently. For example, in contrast to other brain regions, the DG has compensatory mechanisms that occur in response to ischemia, e.g., synaptic plasticity, activation of resident glial cells, neovascularization, and proliferation of stem cells (Nikonenko et al., 2009). In Alzheimer’s disease patients, the DG also exhibits resistance to the accumulation of plaques (Kawles et al., 2023). The fact that the DG is able to undergo neurogenesis (Zhao and van Praag, 2020) and exhibits higher levels of CD31 in vehicle-treated animals compared to cortex and CA1 suggests CD31 has an integral role in EC physiology. Thus, increasing CD31 levels using CMPs may in turn impact neuronal health and neurogenesis. Given the results observed here, future studies exploring the potential therapeutic role of collagen mimetic peptides in promoting neurogenesis and cognitive function are warranted. Finally, our results suggest that CMP treatment promotes vascular-astrocyte interaction at the cellular level, particularly in the CA1 region of the hippocampus (Figure 5). Astrocyte glial cells together with ECs, pericytes, and microglia maintain a tight blood-brain barrier through the formation of a neurovascular unit (Wareham and Calkins, 2020). Our results, although preliminary, suggest that CMP treatment may prove beneficial in preventing neurovascular breakdown, at least in the CA1, which may have therapeutic implications for CNS diseases.

The present study poses intriguing questions warranting further investigation. Collagen is not an inert member of the ECM landscape but instead acts as a key component of a variety of a variety of cellular signaling pathways (Wareham et al., 2024). Breakdown of collagen within the ECM may promote unwanted cellular signaling cascades (e.g., proinflammatory or pro-apoptotic) or cell state changes that lead to tissue degradation. Our fundamental result, that CMP preserved vessel integrity during diabetic insult, likely reflects repair and preservation of structure rather than abject genesis of new vessels. As a hallmark of aging, cellular senescence is a significant contributor to aging and age-related diseases including AD. ECs line the inner wall of blood vessels and are exposed to constant sheer stress from the flow of blood cells (Bloom et al., 2023). ECs located at arterial geometries such as curvatures and branches undergo a higher cell turnover, often becoming senescent (Han and Kim, 2023). Atherosclerosis and diabetes are both diseases associated with hypertension, increasing sheer stress and thus cellular senescence of ECs (Culley and Chan, 2022; Safaie Qamsari and Stewart, 2024). In addition, glycation of collagen type I leads to a premature senescence-like state in endothelial cells (Chen et al., 2002). Senescent cells adopt an inflammatory senescence-associated secretory phenotype (SASP), releasing excessive pro-inflammatory factors such as interleukin 1β (IL-1β), interleukin-6, and MMPs (Han and Kim, 2023). The accumulation of senescent cells in the brain therefore may result in ECM remodeling and collagen breakdown, triggering inflammatory cell activation and subsequent vascular degradation and neurodegeneration (Holloway et al., 2023), which can be accompanied by immune cell infiltration including diapedesis of leukocytes. The longer the experimental period that *ApoE−/−* mice are fed a high-fat diet, the more likely inflammation will exacerbate BBB degradation and immune infiltration.

Taken together, the results here provide a strong foundation for further studies exploring the positive impact of CMP treatment on collagen structure, vessel integrity and survival, and local inflammatory processes. An important question is how CMP repair of endogenous collagen could also influence preservation of other ECM proteins, such as laminin, and of proteins that help anchor cells to the ECM, such as α1β1 and α2β1integrins (Wareham et al., 2024). Such studies will evaluate the potential therapeutic benefit of CMPs in treating and preventing neurodegeneration associated with loss of vessel integrity in the cerebrovasculature. The next steps will include assessing the impact of CMP administration on peripheral vascular pathology due to its prevalence in diabetic patients, as well as on atherosclerotic plaques and associated cerebrovascular disease, diabetic neurodegeneration and cognitive decline. In doing this we will continue to elucidate the potential of CMPs as a novel therapeutic strategy for vascular-related neurodegenerative diseases.

## Data Availability

The raw data supporting the conclusions of this article will be made available by the authors, without undue reservation.
